# Nuclear targeting of the betanodavirus B1 protein via two arginine-rich domains induces G1/S cell cycle arrest mediated by upregulation of p53/p21

**DOI:** 10.1038/s41598-018-21340-x

**Published:** 2018-02-15

**Authors:** Yu-Chin Su, Latif Reshi, Lei-Jia Chen, Wei-Han Li, Hsuan-Wen Chiu, Jiann-Ruey Hong

**Affiliations:** 1 Laboratory of Molecular Virology and Biotechnology, Institute of Biotechnology, National Cheng Kung University, Tainan, 701 Taiwan; 2 Department of Life Science, College of Bioscience & Biotechnology, National Cheng Kung University, Tainan, 701 Taiwan; 3 Department of Biotechnology and Bioindustry, National Cheng Kung University, Tainan, 701 Taiwan

## Abstract

The molecular functions of betanodavirus non-structural protein B and its role in host cell survival remain unclear. In the present study, we examined the roles of specific nuclear targeting domains in B1 localization as well as the effect of B1 nuclear localization on the cell cycle and host cell survival. The B1 protein of the Red spotted grouper nervous necrosis virus (RGNNV) was detected in GF-1 grouper cells as early as 24 hours post-infection (hpi). Using an EYFP-B1 fusion construct, we observed nuclear localization of the B1 protein (up to 99%) in GF-1 cells at 48 hpi. The nuclear localization of B1 was mediated by two arginine-rich nuclear targeting domains (B domain: ^46^RRSRR^51^; C domain: ^63^RDKRPRR^70^) and domain C was more important than domain B in this process. B1 nuclear localization correlated with upregulation of p53 and p21^(wef1/cip1)^; downregulation of Cyclin D1, CDK4 and Mdm2; and G1/S cell cycle arrest in GF-1 cells. In conclusion, nuclear targeting of the RGNNV B1 protein via two targeting domains causes cell cycle arrest by up-regulating p53/p21 and down-regulating Mdm2, thereby regulating host cell survival.

## Introduction

RNA viruses belonging to the Nodaviridae family are classified as Alphanoviruses, which primarily infect insects and Betanoviruses, which predominantly infect fish^1–3^. Betanodaviruses belong to the Betanovirus class and cause virus nervous necrosis (VNN) disease, which is characterized by necrosis of the central nervous system (including the brain and retina), abnormal swimming behavior, darkening of the skin and weight loss^4,5^. Mass mortality caused by VNN in larval and juvenile populations of several teleost species has a significant global economic impact^5^. Betanodaviruses are thought to modulate innate/acquired immunity and may be a useful model for understanding the pathogenesis of RNA virus-mediated diseases.

Nodaviruses are small, non-enveloped, spherical viruses with bipartite positive-sense RNA genomes (RNA1 & RNA2) that are capped but not polyadenylated^3^. RNA1 is the largest genomic segment of the virus and encodes a non-structural protein of approximately 110 kDa, which is designated RNA-dependent RNA polymerase or protein A. This protein is vital for replication of the viral genome. The middle genomic segment, RNA2, encodes a 42-kDa capsid protein that may also function in the induction of cell death^6,7^. RNA3, a sub-genomic RNA species at the 3′ terminus of RNA1, comprises 2 ORFs that encode B1 (a 111 amino acid protein) and B2 (a 75 amino acid protein). The B1 gene of the Red spotted grouper nervous necrosis virus (RGNNV) betanodavirus strain has recently been shown to have an anti-necrotic function during early replication^8^, whereas the B2 gene has been found to either suppress host siRNA silencing^9–11^ or play a role in necrosis.

Many viruses facilitate their own replication by modulating the host cell cycle. DNA viruses, whose primary site of replication is the nucleus, have been studied extensively^12–17^. However, increasing evidence indicates that RNA viruses, whose primary site of replication is normally the cytoplasm, also interfere with the host cell cycle. A number of studies have demonstrated the role of some positive-stranded RNA viruses, such as those belonging to the coronovirus family, during the cell cycle^18–21^. Betanodaviruses comprise the most important positive-stranded aquatic RNA viruses and have caused global concern in the aquaculture industry^4,22^. Increasing outbreaks of betanodavirus infection in grouper fish have resulted in a recent urgent focus on understanding the mechanisms underlying the pathogenesis of betanodavirus infection^11^.

We have previously shown that betanodavirus infection induces cell death and post-apoptotic necrosis in fish cells^7,23,24^. Betanodavirus-induced cell death also correlates with the induction of ER stress and loss of mitochondrial membrane potential in fish cells. RGNNV has recently been shown to induce the production of reactive oxygen species (ROS) during the early and middle replication stages^22^. A number of viral proteins and cell signaling molecules have been shown to be involved in induction of host cell death and post-apoptotic necrosis during betanodavirus infection^7,8,23^. These data suggest that there may be crosstalk between the apoptosis and cell cycle pathways, which share a number of regulatory molecules^24^. We therefore hypothesized that betanodavirus infection may affect the cell cycle in a manner separate from induction of apoptosis.

The present study investigated the mechanisms underlying the 1) targeting of the RGNNV B1 protein into the nucleus and 2) RGNNV-mediated cell cycle modulation in grouper fish cells.

## Results

### Immunofluorescence assay for localization of non-structural protein B1

#### In whole viral infection

**Figure 1:**
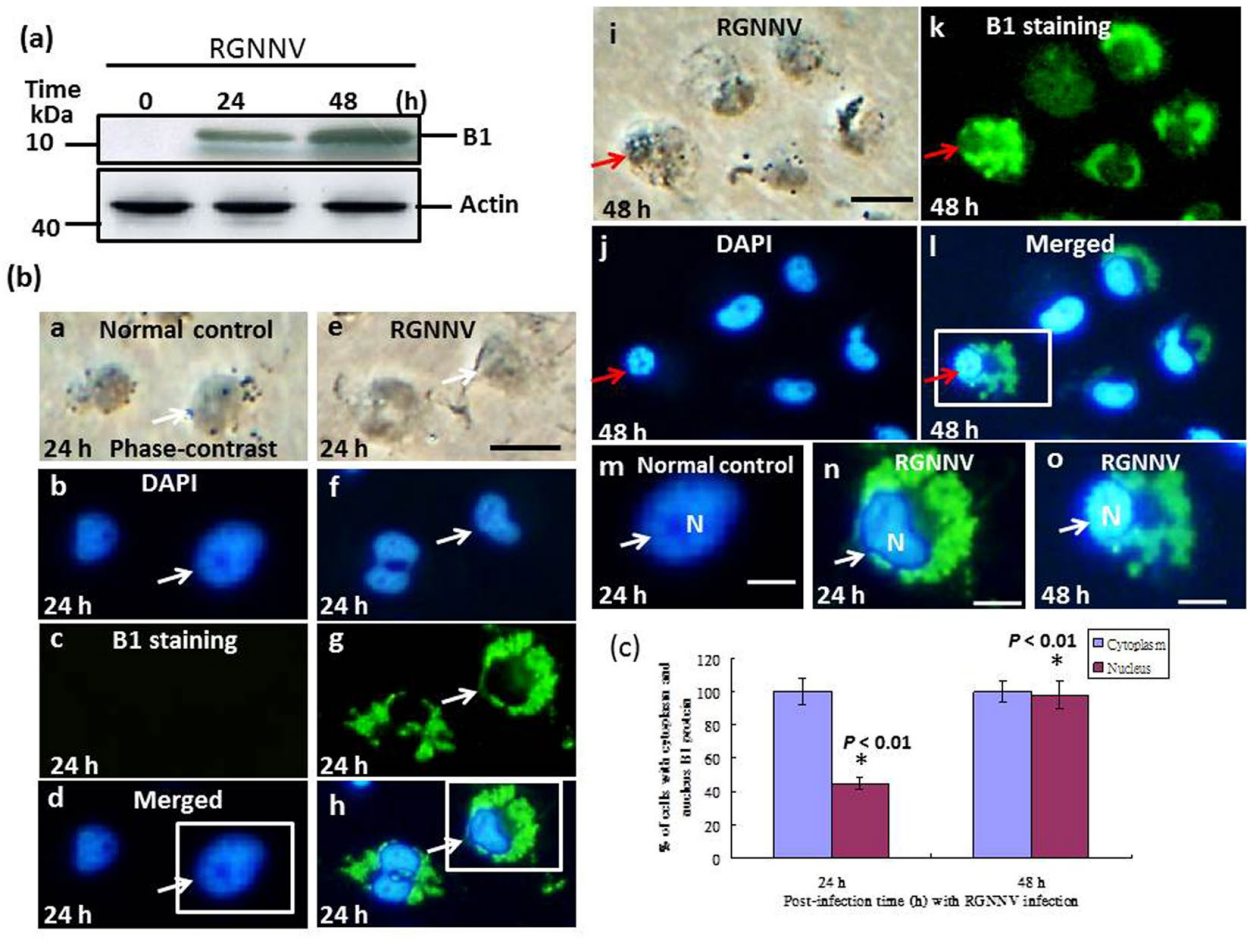
Expression profile of RGNNV B1 protein in cytoplasm and identification of nuclear translocation in RGNNV infection in GF-1 cells. (**a**) Western blotting analysis of B1 protein expression in RGNNV-infected GF-1 cells at different time points. Lanes 1–3 show B1 protein expression at 0 h, 24 h and 48 hpi in RGNNV-infected GF-1 cells. Actin was used as an internal control. (**b**) Immunostaining with B1 antibody and DAPI to determine B1 protein localization in GF-1 cells infected with RGNNV (MOI = 1) at 24 h and 48 hpi. Panels a-d and m (enlarged image) show the normal control; panels e-h and n (enlarged image) show RGNNV-infected GF-1 cells at 24 hpi; panels i-l and o (enlarged image) show RGNNV-infected GF-1 cells at 48 h. Fixed cells were stained with anti-RGNNV protein B1 polyclonal antibody (1:500) and FITC-labeled anti-rabbit secondary antibody. The staining was concentrated in the cytoplasm and only minor staining was observed in the nucleus (panels g, h and enlarged image [n]) at 24 hr; expression was then mainly localized to the nucleus at the 48 hr incubation (panels k, l and enlarged image [o]; indicated by arrows). Samples were stained with DAPI. Panel b (normal control); panel f (RGNNV-infected cells at 24 h); panel j (RGNNV-infected cells at 48 h). Merged images are shown in panel d (normal control); panel h (RGNNV-infected cells at 24 h); and panel l (RGNNV-infected cells at 48 h). Scale bar = 10 μm. (**c**) Quantitative analysis of B1 protein localizations in cytoplasm or targeting into the nucleus by immunofluorescence staining with anti-B1 polyclonal antibody in RGNNV-infected GF-1 cells. The localizations of cytoplasm and nucleus targeting cells were determined in each sample by counting 200 cells. Each result is expressed the mean of 3 independent experiments ± SEM. The data were analyzed using either the paired or unpaired Student’s *t*-test, as appropriate. A value of *p* < 0.05 was considered a statistically significant difference between group mean values.

Western blotting was used to detect the expression of B1 and immunofluorescence assays were used to localize the protein. B1 protein expression was detected in RGNNV-infected cells at 24 hours post-infection (hpi) and continued to increase until 48 hpi (Fig. 1a, lanes 2–3). B1 protein expression in RGNNV-infected cells at 24 hpi was mainly localized to the cytoplasm (100%) partially to the nucleus, in up to 45% of cells (Fig. 1b, e–h; indicated by white arrows; Fig. 1c), whereas at 48 hpi, B1 expression was mainly detected in the cytoplasm (100%) and targeting to nucleus in up to 95% of cells (Fig. 1b, i–l; indicated by the red arrow; Fig. 1c). EYFP-transfected cells were used as a control (Fig. 1c, a–d).

#### In trans-transfection assay using EYFP-B1 fused-genes

**Figure 2:**
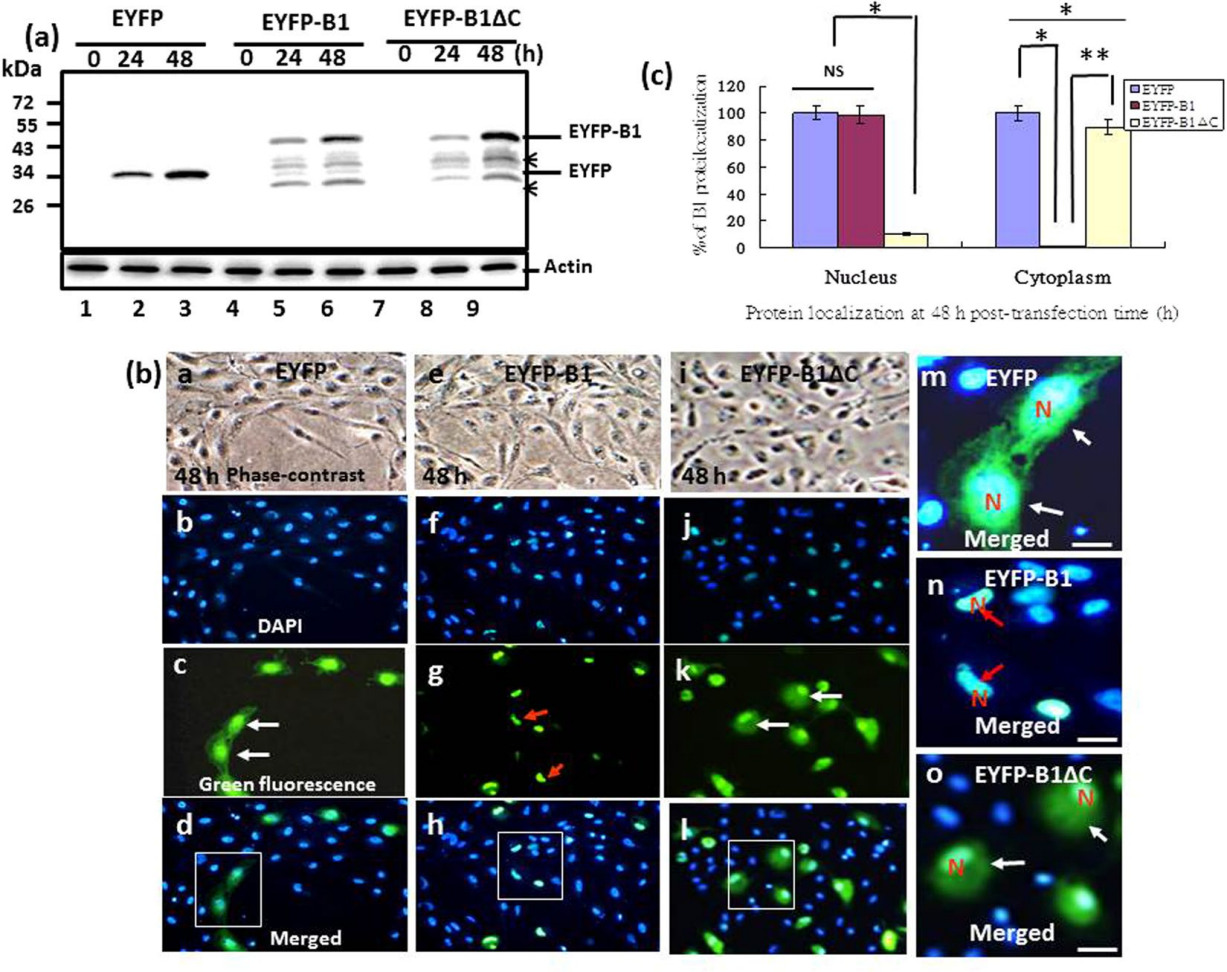
The EYFP-B1 fusion protein is targeted to the nucleus in GF-1 cells at 48 hpt. (**a**) Western blotting analysis showing expression of EYFP, EYFP-B1 and EYFP-B1ΔC fusion proteins detected with an anti-EYFP monoclonal antibody at 0 h, 24 h and 48 hpt (lanes 1–3 show the EYFP-transfected group, lanes 4–6 show the EYFP-B1 fusion protein group and lanes 7–9 show the EYFP-B1ΔC fusion protein group). Actin was used as the internal control. (**b**) Phase-contrast fluorescence images showing the distribution of EYFP and EYFP-B1 in transfected GF-1 cells at 48 hr. Panels e-h show EYFP-B1 distributed in the nucleus. EYFP control cells are shown in panels a-d and EYFP-B1ΔC negative control cells are shown in panels i–l. The magnified images in panels m (EYFP), o (EYFP-B1ΔC) and n (EYFP-B1) show EYFP-B1 distributed in the nucleus in the EYFP-B1 samples (indicated by red arrows). N: nucleus. Scale bar = 10 μm. (**c**) Quantitative analysis of B1 protein targeting to the nucleus by EYFP direct monitoring system as constructed with the EYFP-B1 fusion genes by transfection with different plasmids into GF-1 cells at 48 h post-transfection time. The nucleus targeting cells were determined in each sample by counting 200 cells. Each result is expressed as the mean of 3 independent experiments ±SEM. The data were analyzed using ANOVA with multiple comparisons, as appropriate. A value of *p < 0.01 and **p < 0.05 was considered a statistically significant difference between mean group values.

The EYFP, EYFP-B1 and EYFP-B1ΔC (for details see Methods) fusion constructs were used to directly trace B1 protein targeting to the nucleus. Western blot analysis was used to confirm the expression of the EYFP-B1 fusion protein (Fig. 2a, lanes 4–6), the EYFP-B1ΔC fusion protein (Fig. 2a, lanes 7–9) and the EYFP protein (Fig. 2a, lanes 1–3) in cells harvested at 0 h, 24 h and 48 hours post-transfection (hpt). A degraded form of the protein is labeled by arrows in the figure. DAPI staining revealed that although the EYFP-B1 fusion protein was targeted to the nucleus (Fig. 2b: e, f, g, h and n [enlarged image]), this targeting was not observed for EYFP alone (Fig. 2b: a, b, c, d and m [enlarged image]). Loose nucleus targeting was observed for the EYFP-B1ΔC fusion protein (Fig. 2b: i, j, k, l and o [enlarged image]), which was distributed throughout the whole cell, including the cytoplasm and nucleus at 48 hpt. In the nucleus targeting test, in EYFP-B1ΔC fusion protein also lost its targeting ability (Fig. 2b, o), as compared with that of EYFP-B1 (Fig. 2b, n) and EYFP alone (Fig. 2b, m). Quantitative analysis of the EYFP, EYFP-B1 and EYFP-ΔB1 proteins targeting into cytoplasm or nucleus (Fig. 2c) showed that GF-1 cells transfected with pEYFP, pEYFP-B1 and pEYFP-B1ΔC plasmids exhibited a significant targeting into the nucleus in EYFP-B1-transfected cells (up to 99%) at 48 hpt (*p < 0.01; **p < 0.05) compared with cells transfected with EYFP (100%) and EYFP-B1ΔC plasmids (10%), which apparently lost targeting ability. The reasons for these observations are discussed below.

### Identification of two arginine-rich nuclear targeting domains in the B1 protein

**Figure 3:**
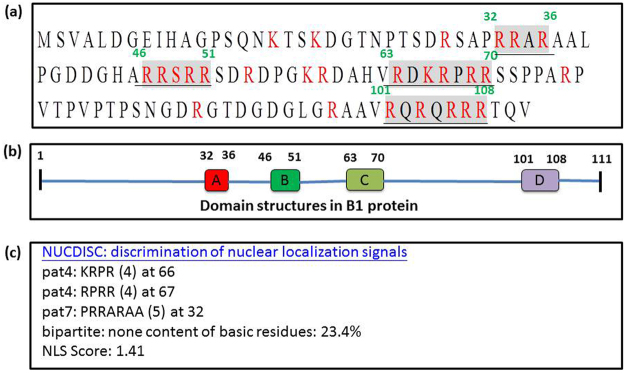
Prediction of nuclear targeting domains in the RGNNV B1 protein. (**a**) The complete amino acid sequence of B1 protein (111 amino acids) and (**b**) four possible functional domains of the B1 protein. Domain design was based on program prediction. (**c**) Domain A starts from amino acid 32 to 36; Domain B starts from amino acid 46 to 51; Domain C starts from amino acid 63 to 70; Domain D starts from amino acid 101 to 108.

**Figure 4:**
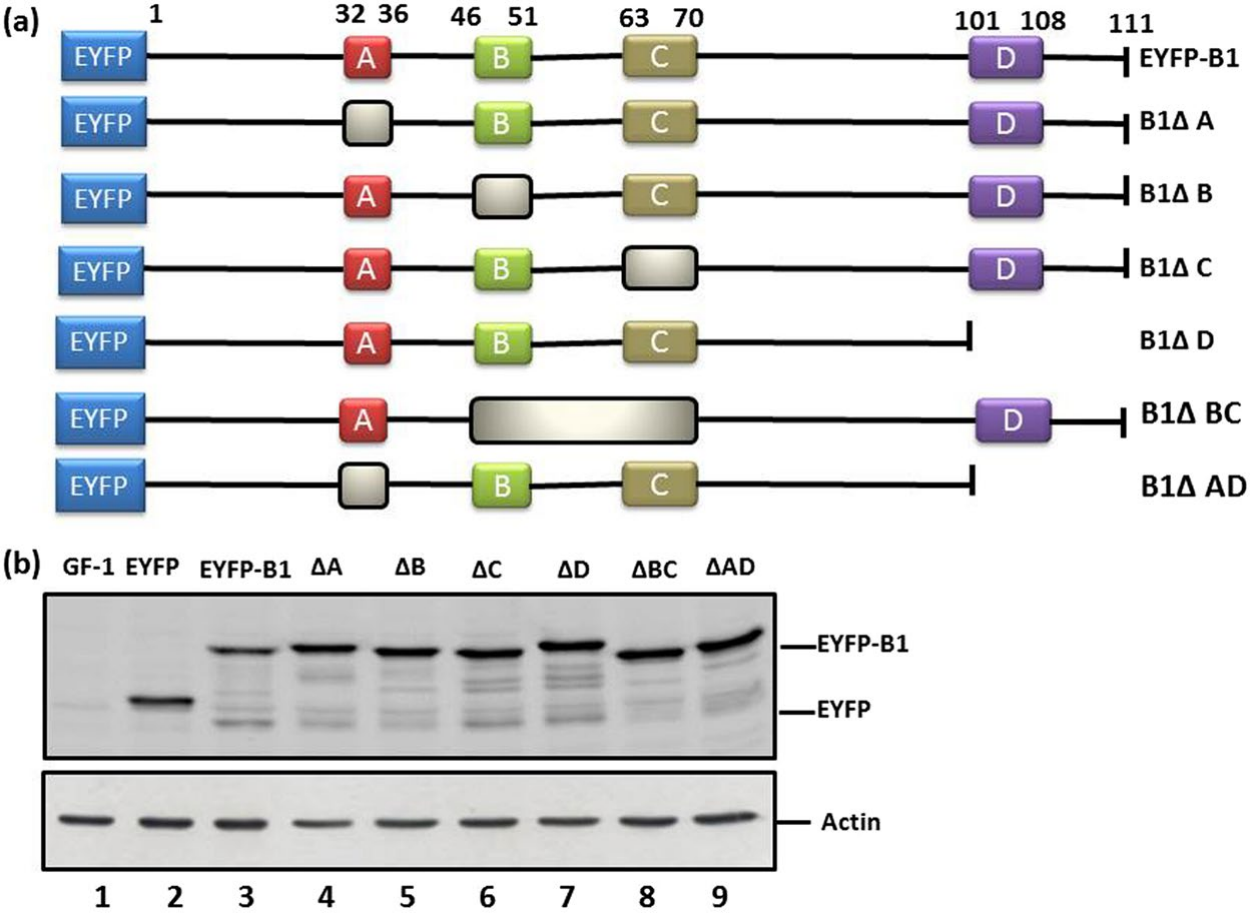
Design and construction of the wild type B1 and different domain deletion mutants of B1. (**a**) The solid squares represent the domains and the empty boxes represent the deleted domains. A total of 7 domain deletion mutants were constructed. (**b**) Expression of EYFP-B1 and different mutant forms evaluated by western blot analysis of GF-1 cells at 24 hpt. Lane 1, negative control GF-1 cell lysate; lane 2, EYFP alone; lane 3, EYFP-B1; lanes 4–7, single domain deletions including B1ΔA, B1ΔB, B1ΔC and B1ΔD; lanes 8–9; double domain deletion mutants including B1ΔBC and B1ΔAD. Actin was used as an internal control.

**Figure 5:**
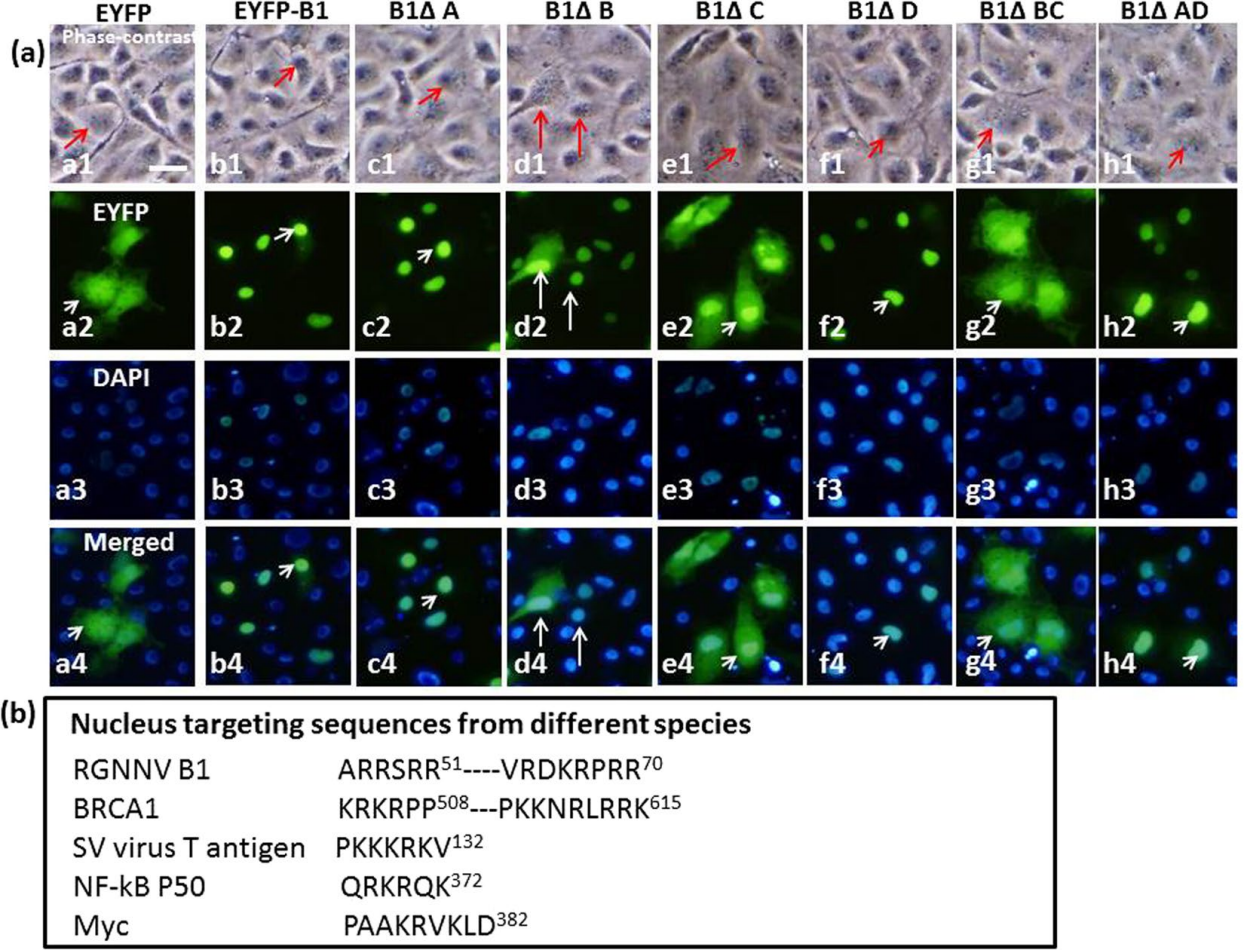
Fluorescence microscopy and DAPI staining confirm the role of domains B and C in nuclear targeting of B1 in GF-1 cells. (**a**) Phase-contrast fluorescence images showing the distribution of EYFP, EYFP-B1 and different B1 domain deletion mutants in transfected GF–1 cells at 48 hpt. Panels a1–a4 show EYFP distributed in the cytoplasm; panels b1–b4 show EYFP-B1 distributed in the nucleus; panels c1–c4 show EYFP-B1ΔA distributed in the nucleus; panels d1–d4 show EYFP–B1ΔB distributed in the cytoplasm (major) and nucleus (minor; indicated by arrows); panels e1–e4 show EYFP–B1ΔC distributed in the cytoplasm (indicated by arrows); panels f1–f4 show EYFP–B1ΔD distributed in the nucleus; panels g1–g4 show EYFP–B1ΔBC distributed in the cytoplasm; panels h1–h4 show EYFP–B1ΔAD distributed in the nucleus. The cells were also traced with green fluorescence and DAPI nuclear staining and the final images were merged to confirm localization. Scale bar = 10 μM. (**b**) Comparison of different nuclear targeting signal sequences.

Potential targeting signal peptides in the B1 protein were identified by running the iPSORT and TargetP 1.1 programs (Fig. 3) for the R and K residues. The predicted targeting domains were labeled domain A (^32^PRRAR^36^), domain B (^46^ARRSRR^51^), domain C (^63^VRDKRPRR^70^) and domain D (^101^VRQRQRRR^108^) (Fig. 3). Domain deletion experiments were used to determine which targeting domain(s) played a role in the nuclear targeting of B1 (Fig. 4a). Western blot experiments were used to compare the localization of the EYFP-B1 fusion protein (~40-kDa) with (1) domain A deletion (<40-kDa, designed as ΔA), (2) domain B deletion (ΔB), (3) domain C deletion (ΔC), (4) domain D deletion (ΔD), (5) domain A and D double deletion (ΔAD) and (6) domain B and C double deletion (ΔBC) (in Fig. 4b, lanes 3–9, respectively). Untransfected GF-1 cells and EYFP-transfected cells were used as negative controls (Fig. 4b, lanes 1–2, respectively). These data, along with nuclear targeting analysis (Fig. 5a; Table 1) showed that deletion of domain C resulted in the biggest decrease in nuclear targeting (in ΔC panel: panels e1-e4; indicated by arrows) and deletion of domain B resulted in a minor decrease in nuclear targeting (panels d1-d4; indicated by arrow). In contrast, deletion of domain A (panels c1-c4) and domain D (panels f1-f4) had no significant effect on nuclear targeting. Simultaneous deletion of two domains (B1ΔBC; panels g1-g4 and B1ΔAD; panels h1-h4) (Table 1) also showed that domains B and C played an important role in the nuclear targeting of B1, whereas domains A and D were not involved in the nuclear targeting of B1.

**Table 1:** Identification of the different proteins distribution with EYFP monitoring in fish cells.

Constructions	Localizations	
	Nucleus	Cytoplasm
EYFP	++	+++
EYFP-B1	+++	—
ΔA	+++	—
ΔB	++	+
ΔC	++	+++
ΔD	+++	—
ΔBC	+	+++
ΔAD	+++	—

### Nuclear targeting of the B1 protein is correlated with an induction of G1/S cell cycle arrest in GF-1 cells

**Figure 6:**
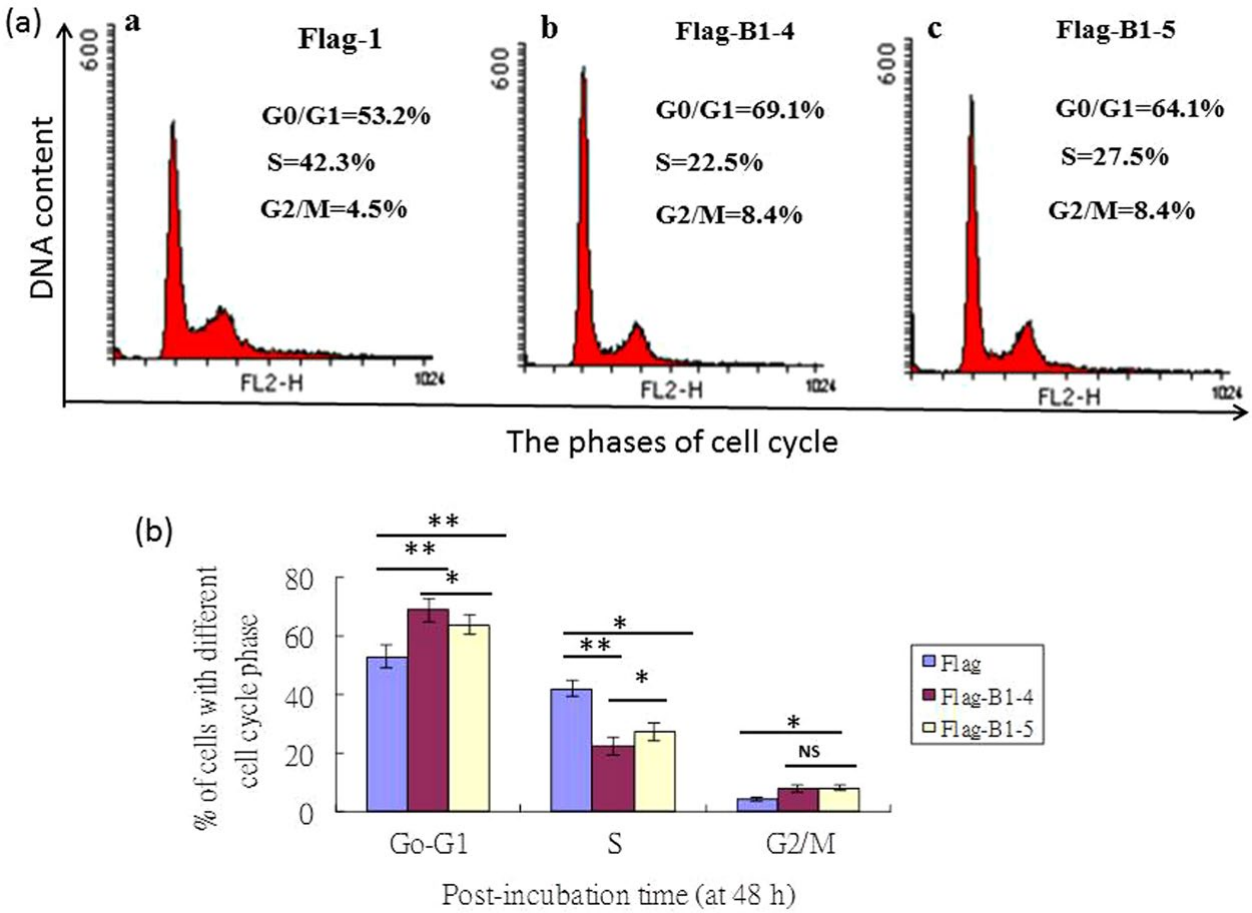
Nuclear targeting of the RGNNV B1 protein is correlated with G1/S cell cycle arrest in GF-1 cells at 48 hpt. The Flag-1, Flag-B1-4 and Flag-B1-5-producing GF-1 cells were fixed, stained with PI and analyzed by flow cytometry. (**a**) Cell cycle profiles of Flag-1, Flag-B1-4 and Flag B1-5 at 48 hpt. (**b**) Analysis of cell cycle phase distribution Flag-1 (as negative control), Flag-B1-4 and Flag-B1-5-producing GF-1 cells at 48 hpt. Each result is expressed the mean of 3 independent experiments ±SEM. Data were analyzed using ANOVA with multiple comparisons, as appropriate. A value of *p < 0.01 and **p < 0.05 was considered a statistically significant difference between mean group values.

We have previously shown that anti-sense RNA-mediated decreases in B1 protein expression decrease the viability of RGNNV-infected host cells^8^. In the present study, we investigated whether B1 plays a role in cell division during a specific phase of the cell cycle. Cells transfected with pFlag and pFlag-B1 and selected for receiving the different producing cell line as term Flag-1 for negative control and Flag-B1-4 and Flag-B1-5 for B1-producing cell lines. These cells were subjected to cell cycle analysis at 48 hpt by measurement of DNA content by using PI staining. Representative cell cycle profiles and histograms of Flag-1, Flag-B1-4 and Flag-B1-5 cells are presented in Fig. 6a and b, r*e*spectively. There was also a significant increase in the percentage of cells arrested at the G0/G1/phase in the Flag-B1-4 and Flag-B1-5 samples compared with the Flag-1 sample (69.1% vs. 64.1% vs. 53.2%, respectively) (*p < 0.01). There was a significant decrease in the percentage of cells arrested at the S phase in Flag-B1-4 and Flag-B1-5 samples compared with the Flag-1 sample (22.5% vs. 27.5% vs. 42.3, respectively). In contrast, there was a minor significant increase in the percentage of cells in the G2/M phase among the three samples (8.4% vs. 8.4% vs. 4.5%, respectively) (*p < 0.01; **p < 0.05). To summary B1 protein can arrest cell cycle progression at G1/S phase.

### B1-induced G1/S cell cycle arrest was correlated with upregulation of p53 and p21 and downregulation of Cyclin D1, CDK 4 and Mdm2

**Figure 7:**
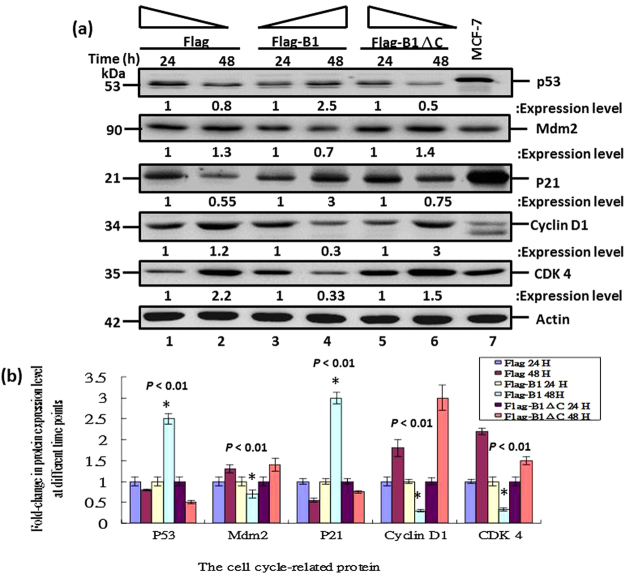
B1 protein-induced G1/S cell cycle arrest was correlated with upregulation of p53 and p21 in GF-1 cells. (**a**) GF-1 cells were transfected with Flag, Flag-B1 and Flag-B1ΔC plasmids and cell lysates were collected at the indicated time points. Cellular proteins were detected by western blot analysis and protein amounts were standardized to that of actin. Expression of several proteins involved in the G0/G1-to-S-phase transition was analyzed, including Cyclin D1, CDK-4, p21 and p53. Lanes 1–2 show cells transfected with Flag alone at 24 h and 48 hpi, respectively; Lanes 3–4 show cells transfected with Flag-B1 at 24 h and 48 hpi, respectively; Lanes 5–6 show cells transfected with Flag-B1ΔC at 24 h and 48 hpi, respectively; Lane 7 shows human MCF-7 cell lysate as a positive control. Actin is shown as a loading control. The experiment was repeated three times and representative blots are shown. (**b**) Quantitative analysis of p51, Mdm2, p21, Cyclin D1 and CDK 4 protein expression in Flag-transfected cells, Flag-B1-transfected cells and Flag-B1ΔC-transfected cells. The results presented are average values obtained from three different experiments with bars indicating standard deviation. Data were analyzed using either paired or unpaired Student’s t-tests, as appropriate. A value of *p* < 0.05 was considered a significant difference between group mean values.

The G1/S transition is regulated by a number of molecules including the cyclin-Cdk complexes, pRb, Mdm2 and CKIs such as p21^25^. We investigated the key molecules responsible for RGNNV B1-induced cell cycle arrest by examining the expression profiles of host G1/S transition proteins. GF-1 cells were transfected with Flag, Flag-B1 and Flag-B1ΔC plasmids and then assayed for expression of these cellular proteins by western blotting at 24 hpt and 48 hpt. There was a significant increase in the expression of p53 and p21 (Fig. 7a; lanes 3 and 4) in GF-1 cells transfected with B1 plasmids compared with cells transfected with Flag (Fig. 7a; lanes 1 and 2) and Flag-B1ΔC (Fig. 7a; lanes 5 and 6) plasmids at 48 hpt. In contrast, there was a significant downregulation of the expression of Cyclin D1, CDK4 and Mdm2 (Fig. 7a; lanes 3 and 4) in GF-1 cells transfected with B1 plasmids compared with cells transfected with Flag (Fig. 7a; lanes 1 and 2) and Flag-B1ΔC (Fig. 7a; lanes 5 and 6) plasmids at 48 hpt. Quantitative analysis of the expression levels relative to the actin loading control showed that GF-1 cells transfected with Flag-B1 plasmids exhibited a significant increase in the expression of p53 and p21 at 48 hpt (p < 0.01) and a significant decrease in the expression of Cyclin D1, CDK4 and Mdm2 at 48 hpt (p < 0.01) compared with cells transfected with Flag and Flag-B1ΔC plasmids.

## Discussion

Our present study identified a 7 amino-acid arginine-rich signal peptide (RDKRPRR) that targeted the non-structural RGNNV B1 protein to the nucleus of infected GF-1 cells. Our data suggested that the B1 protein affects cell survival, because nuclear targeting of B1 correlated with upregulation of p53 and p21 and downregulation of Cyclin D1, CDK4 and Mdm2, thus resulting in G1/S cell cycle arrest. Our data may provide some insight into the molecular mechanisms involved in the pathogenesis and disease control in RGNNV infections.

### Nuclear targeting of B1 protein

The B1 and B2 proteins have previously been shown to be encoded by the sub-genomic alphanovirus RNA3 synthesized from the 3′ terminus of RNA1^26–28^. Synthesis of B1 protein has been confirmed experimentally only for FHV^28^ and the subcellular localization and functions of B1 have not been widely studied. In this study, we examined B1 expression patterns and investigated the role of B1 in cell cycle regulation in RGNNV-infected fish cells.

Our data showed that B1 mRNA was expressed in RGNNV-infected cells between 12 h and 24 hpi and the levels increased rapidly until 48 hpi. Although both domain B (^46^RRSRR^51^) and domain C (^63^RDKRPRR^70^) played a role in nuclear targeting of B1 in RGNNV-infected GF-1 cells, domain C had a more significant targeting role, which was probably mediated via its arginine-rich structure. Our data were consistent with previous results showing that nuclear targeting of the BRCA1 protein is mediated by two domains, in contrast to the SV40 virus T antigen, NF-kB and Myc proteins^29^. It will be interesting to investigate the role of importin 1 in the nuclear transport of B1.

### Role of B1 protein in cell cycle progression

Apoptosis and necrosis are two major mechanisms mediating cell death in nucleated eukaryotic cells^30,31^. Betanodavirus infection has been shown to induce host cell death and post-apoptotic necrosis in fish cells^7,8,23^. Many viruses interfere with the host cell cycle and thereby achieve high replication efficiencies and virus yields^24^. Because viral protein expression and production of progeny virus is predominantly observed in the G1/S interface, cell cycle arrest at that point appears to be a common event observed in cells infected with different viruses of various strains and subtypes. We used flow cytometry analysis to show that the B1 protein induced G1/S cell cycle arrest in RGNNV-infected GF-1 cells and immunoblotting data confirmed a downregulation of Cyclin D1, CDK4 and Mdm2 expression in GF-1 cells transfected with the Flag-B1 vector. The G1/S cell cycle arrest was mediated predominantly via domain C.

The role of the Rb-E2F pathway in cell cycle progression out of G0 through G1 and into the S-phase^32^ makes it a suitable target for viruses^33,34^. Maintenance of Rb proteins in an activated (hyper phosphorylated) state induces cell cycle arrest at the G0 or G1 phase or at the G1/S boundary, thus preventing host DNA replication during viral lytic infection^15,35,36^. Virus-induced modulation of the host cell cycle has also been shown to be beneficial during viral replication, transcription, translation and assembly^37–41^. Previous data have shown that cell cycle arrest induced by enveloped RNA viruses can favor viral assembly, because the endoplasmic reticulum (ER) and Golgi apparatus disassemble into vesicles and larger membrane structures break into clusters or remnants during mitosis^42–44^. It is therefore possible that RGNNV-induced cell cycle arrest may prevent early death of infected cells^7,8,23^. Because a delay in apoptotic/necrotic cell death always occurs after cell cycle arrest^25,45,46^, it is possible that RGNNV-induced G1/S cell cycle arrest may inhibit early apoptosis of infected cells to gain sufficient time and resources for the viral life cycle to produce new viral progeny.

### B1 protein induces p53 expression and decreases Mdm2 expression

The role of p21 and p53 in the regulation of cell cycle progression through the G1/S phase is well documented^25,46,47^. Our present study showed a B1-mediated upregulation in the expression of p21 and p53 in GF-1 cells at 48 hpt. Although the mechanism underlying this induction is unknown, it is likely to be mediated through ROS signals in GF-1 cells^48–50^.

P21, which is regulated by p53, inhibits the formation of the Cyclin D-CDK4/6 complex, which is important for the G1/S transition and inhibits Mdm2 as a P53 inhibitor^51^. Interestingly, our data also showed a B1-mediated decrease in the expression levels of both Cyclin D1 and CDK4, thus suggesting that cell cycle arrest in RGNNV-infected GF-1 cells may be mediated via inhibition of B1-mediated direct inhibition of mRNA transcription or stability and/or translation of Cyclin D1 and CDK4. Our data suggested that B1-mediated inhibition of Cyclin D1/CDK4 complex formation, Mdm2, P53 inhibitor and G1/S cell cycle arrest occur via multiple pathways. Moreover, we found that betanodavirus infected the B1-producing GF-1 cells, thereby decreasing host cell viability by approximately 40% at 48 hpt compared with wild type GF-1 cells^8^.

**Figure 8:**
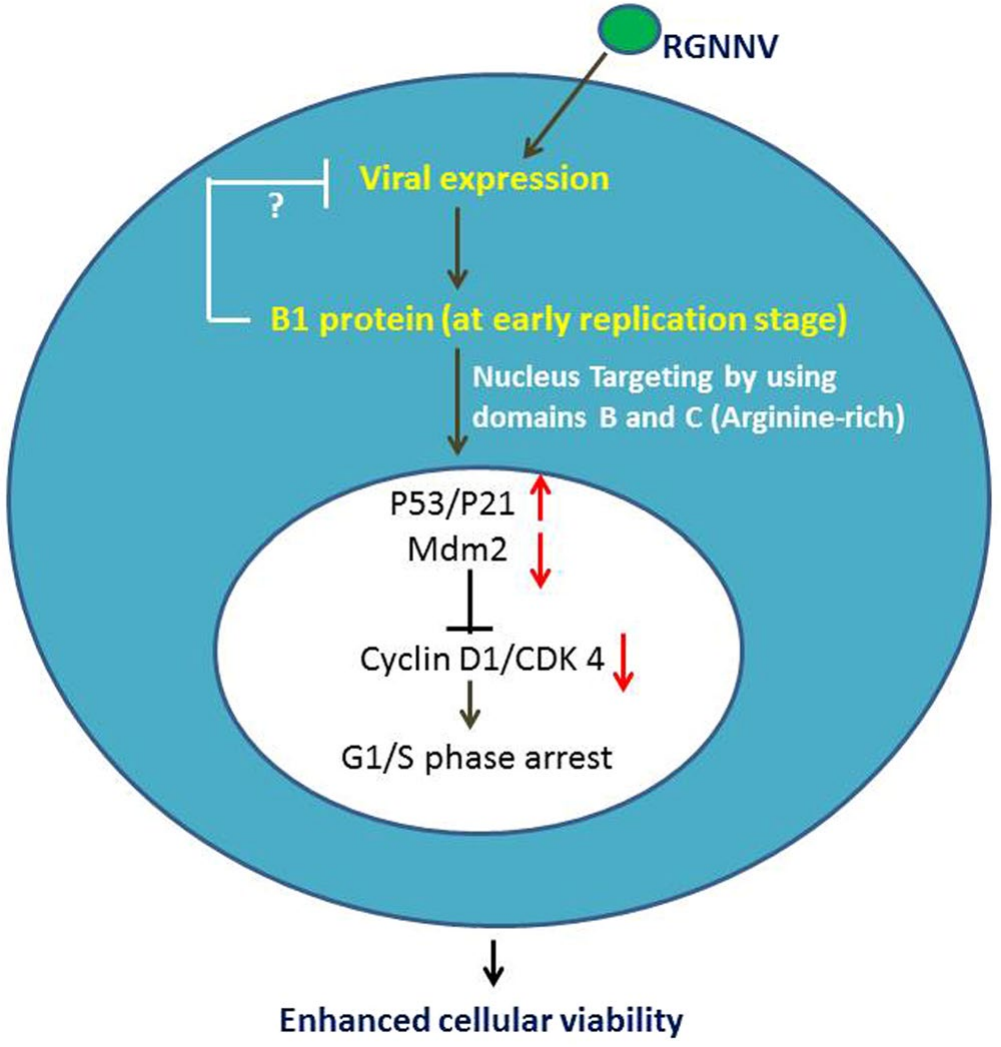
Hypothesis explaining the mechanism by which nuclear targeting of the RGNNV B1 protein via domains B and C induces p53/p21-mediated cell cycle arrest in GF-1 cells. The novel RGNNV non-structural protein B1 is expressed at an early replication stage (between 12 hpi and 24 hpi) in GF-1 cells. EYFP tagging shows the nuclear localization of B1 at 24 h (minor) and 48 hpi (mainly). Prediction of nuclear localization signals and domain deletion experiments showed that domains B and C, but not domains A and D, play a role in nuclear targeting. Nuclear targeting of the B1 protein induced cell cycle arrest of GF-1 cells at the G1/S phase via upregulation of p21 and p53 expression and downregulation of Cyclin D1, CDK4 and Mdm2 expression at 48 hpt.

In summary (Fig. 8), we found that the RGNNV non-structural protein B1 was expressed during early replication (between 12 h and 24 hpi) in GF-1 cells. B1 was targeted to the nucleus at 48 hpi. Domain deletion experiments were used to identify the arginine-rich domain C, which was found to play a major role in the nuclear localization of B1. Nuclear targeting of the B1 protein was correlated with cell cycle progression, B1-mediated cell cycle arrest at the G1/S interphase and decreased cell volume at 48 hpt. B1 protein-induced cell cycle arrest was correlated with upregulated expression of p21 and p53 and downregulation expression of the Cyclin D1, CDK4 and Mdm2 proteins. Our findings may provide new insights into the mechanisms underlying the pathogenesis of RGNNV infection. Our findings provide new insights into RNA viral protein interactions with host proteins.

## Methods

### Cell lines and Virus

The GF-1 grouper cell line was provided by Dr. Chi (Institute of Zoology and Development of Life Sciences) and was cultured at 28 **°**C in Leibovitz L-15 medium supplemented with 5% fetal bovine serum and 25 µg/ml gentamycin. GF-1 cells were infected with RGNNV obtained from naturally infected red grouper larvae collected in 2002 in the Tainan prefecture. The virus was purified as previously described^52^ and stored at −80 **°**C until use.

### Immunofluorescence Assay

#### For whole virus infection and traced B1 protein assay

The cells were infected for 24 h or 48 h with RGNNV (MOI = 1), rinsed once with PBS, fixed with 4% paraformaldehyde for 15 min. at room temperature and then permeabilized for 10 min with 0.2% Triton X-100 in PBS at room temperature. For the immunofluorescence assay, the cells were incubated with a 1:50 dilution of primary polyclonal antibody against RGNNV protein B1 for 1 hr at room temperature. Cells were washed with PBST (0.05% Tween-20) and then incubated with a 1:100 dilution of secondary antibody conjugated to TRITC or fluorescein isothiocynate (FITC-conjugate goat anti-rabbit IgG) for 40 min. at room temperature. Cells were washed 3 times with PBST and examined using an Olympus 1 × 70 fluorescence microscope equipped with 488-nm excitation and 515 nm long pass filter detection. DAPI counterstaining was performed according to the manufacturer’s instructions^23^.

#### For EYFP-B1 fused genes assay

GF-1 cells cultured on 35-mm plates were transfected with 2 µg pEYFP, pEYFP-B1 or pEYFP-B1ΔC using Lipofectamine plus (Life Technologies-USA) according to the manufacturer’s instructions, after which cells were analyzed by fluorescence microscopy using 488 nm excitation and a 515 nm long-pass filter^23^.

### Construction of B1 plasmids, domain deletion mutants and cell transfection

The 336-nt RGNNV B1 gene was cloned from the RGNNV genome as previously described^8^. PCR products were sequenced by dye termination method using an ABI PRISM 477 DNA sequencer (PE Biosystems, Foster City, CA, USA). The sequences were scanned against the GenBank database BLAST (www.genome.jp/tools/clustalw/) and the SBASE domain prediction system (www.icgeb.trieste.it/sbase/) programs^8^.

The B1 encoding sequence^8^ and all deletion fragments were cloned into pcDNA3.1 (Clontech, USA) or p3XFLAG-*myc*-CMV-26 vectors (Sigma) or into the pEYFP-C1 vector (Clontech) in-frame with the EYFP and sequenced to verify the reading frame. The primers and restriction enzymes sites used to construct the different recombinant plasmids are shown in Table 2. The recombinant plasmids were amplified by taq/pfu DNA polymerase with the designed primers. The PCR products were digested with *Eco*RI and *Bgl*II or *Bam*HI at 37 °C for 3 h and ligated with the appropriate vectors to create pEYFP-B1, pEYFP-B1ΔA, pEYFP-B1ΔB, pEYGP-B1ΔC, pEYFP-B1ΔD, pEYFP-B1ΔBC and pEYFP-B1ΔAD (Fig. 4). For cell transfections, GF-1 cells were seeded in 60-mm culture dishes (3 × 10^5^ cells/dish) 1 day before the transfection procedure. The cells were transfected with 2 μg of the recombinant plasmid mixed with Lipofectamine 2000 (Invitrogen, USA) according to the manufacturer’s instructions.

**Table 2:** The RGNNV B1 sequence primers used in this study.

Name		Primers*
pEYFP-B1ΔA	Forward 1	AGCTAAGCTTCGATGTCTGTCGATTAGAC (HindIII)
	Reverse 1	AATTGAATTCAGGTGCTGATCGATCGCT (EcoRI)
	Forward 2	AATTGAATTCGCAGCTCTTCCAGGTGAT (EcoRI)
	Reverse 2	GATCGGATCCCTACACTTGAGTGCGACG (Stop)
pEYFP-B1ΔB	Forward 1	AGCTAAGCTTCGATGTCTGTCGATTAGAC (HindIII)
	Reverse 1	AATTGAATTCCGCGTGTCCATCATCACC (EcoRI)
	Forward 2	AATTGAATTCAGTGACCGCGATCCAGGT (EcoRI)
	Reverse 2	GATCGGATCCCTACACTTGAGTGCGACG (Stop)
pEYFP-B1ΔC	Forward 1	AGCTAAGCTTCGATGTCTGTCGATTAGAC (HindIII)
	Reverse 1	AATTGAATTCAACGTGCGCATCTCGTTT (EcoRI)
	Forward 2	AATTGAATTCAGCTCGCCGCCTGCACGT (EcoRI)
	Reverse 2	GATCGGATCCCTACACTTGAGTGCGACG (Stop)
pEYFP-B1ΔD	Forward 1	AGCTAAGCTTCGATGTCTGTCGATTAGAC (HingIII)
	Reverse 1	GATCGGATCCCTACACAGCAGCTCGGCCTAG (Stop)
pEYFP-B1ΔBC	Forward 1	AGCTAAGCTTCGATGTCTGTCGATTAGAC (HindIII)
	Reverse 1	AATTGAATTCCGCGTGTCCATCATCACC (EcoRI)
	Forward 2	AATTGAATTCAGCTCGCCGCCTGCACGT (EcoRI)
	Reverse 2	GATCGGATCCCTACACTTGAGTGCGACG (Stop)
pEYFP-B1ΔAD	Forward 1	AGCTAAGCTTCGATGTCTGTCGATTAGAC (HindIII)
	Reverse 1	AATTGAATTCAGGTGCTGATCGATCGCT (EcoRI)
	Forward 2	AATTGAATTCGCAGCTCTTCCAGGTGAT (EcoRI)
	Reverse 2	GATCGGATCCCTACACAGCAGCTCGGCCTAG (Stop)

^*^The enzymatic cutting sites on nucleotides of primers are underlined.

### Western Blot Analysis

GF-1 cells stably transfected with EYFP, EYFP-B1 or EYFP-B1ΔC were seeded in 60-mm dishes at a density of 9 × 10^5^ cells/dish. The cells were incubated for 0, 24 and 48 h after which the culture medium was aspirated and the cells were washed with 1% PBS and then lysed with lysis buffer (10 mM Tris, 20% Glycerol, 10 mM SDS, 2% B-mercaptoethanol, pH-6.8). The cell lysates were separated on SDS polyacrylamide gels to resolve the proteins and transferred to nitrocellulose (NC) membranes. The membranes were immunoblotted with primary antibodies (1:500 for B1 polyclonal antibodies, 1:2500 for actin antibodies and 1:10,000 for EYFP monoclonal antibodies) as previously described^53^. The membranes were washed and incubated with 1:5000, 1:2500 and 1:10,000 dilutions of peroxide-labeled goat anti-rabbit or mouse conjugated antibodies, respectively. Protein expression was detected with chemiluminescence and the signal was captured by using the Top Bio Multigel-21 system (Total Lab Systems TLS).

### Selection of stable RGNNV B1-overexpressing cell lines

Flag-producing cells (negative control) and Flag-B1–producing cells were obtained by transfection of GF-1 cells with pFlag and pFlag-B1, respectively, was cloned by Dr. Su^8^ using Lipofectamine-Plus (Life Technologies, Grand Island, NY, USA) according to the manufacturer’s instructions and positive clones were selected with G418 (800 mg/ml; Sigma Chemical, MO, USA). Transcription of the inserted coding sequences in these vectors is driven by the immediate-early promoter of human cytomegalovirus. Selection time (2.5–3 months) from a single colony varied depending on properties.

### Cell cycle analysis by flow cytometry

Cell cycle distribution and nuclear DNA content were determined by propidium iodide (PI) staining using flow cytometry as described previously^54^. Briefly, the cells were trypsinized, washed once with PBS and fixed with 75% ethanol for 1 hr at 20 **°**C. Fixed cells were washed with PBS and incubated for 1 hr at room temperature with 1 ml PBS containing 2 µg/ml PI stain, Triton X 0.1% and RNase 0.2 µg/ml. Each data point (10,000 cells) represents the mean viability of 3 independent experiments ±SEM. Data were analyzed using ANOVA with multiple comparisons as appropriate. A value of *p < 0.01; **p < 0.05 was considered a statistically significant difference between mean values of groups.

### Western blotting analysis to analyze proteins involved in cell cycle progression

The expression of cell cycle regulatory molecules such as Cyclin D1, CDK4, Mdm2, p53 and p21 was analyzed by western blotting to investigate the molecular mechanisms of B1-induced G1/S cell cycle arrest. Cells were harvested at 24 and 48 hr after transfection, washed once with PBS and then lysed directly in lysis buffer (RIPA-Tris HCL: 50 mM, pH 7.4, NP40: 1%, Na-deoxycholate: 0.25%, NaCl: 150 mM, EDTA: 1 mM + Protease inhibitors). The lysates were then boiled at 96 **°**C for 10 min. Whole cell lysates were separated by SDS-PAGE and the proteins transferred to NC membranes and immunoblotted with the relevant primary and secondary antibodies. The antibodies used for western blotting included anti-cyclin D1, anti-CDK4 and anti-P21 (all from Gene tek) and anti-p53 and anti-ß-actin (Millipore). Primary antibodies were used at dilutions of 1:3000 (CDK4 and Cyclin D1 polyclonal antibodies), 1:600 (P21 polyclonal antibody), 1:1000 (p53 polyclonal antibody) and 1:12500 (actin monoclonal antibody). The membranes were then washed and incubated with 1:7500, 1:1000 and 1:12500 dilutions of anti-mouse or rabbit conjugated secondary antibodies, respectively. Antibody binding was detected by chemiluminescence and the signal was captured with a Top Bio Multigel-21 imaging system (Total Lab Systems TLS)^23^.

### Statistical analysis

The percentages of B1 protein localized in cytoplasm or targeting to the nucleus with RGNNV infection in GF-1 cells at 0 h, 24 h and 48 hpi. The localizations of cytoplasm and nucleus targeting cells were determined in each sample by counting 200 cells. Each result is expressed as the mean of 3 independent experiments ±SEM. The data were analyzed using either the paired or unpaired Student’s *t*-test or ANOVA with multiple comparisons as appropriate. Thresholds for significance are indicated on each figure. A value of *p* < 0.05 was considered a statistically significant difference between group mean values.
